# Ustekinumab is effective against ulcerative colitis with intestinal stenosis: A case report

**DOI:** 10.1097/MD.0000000000031213

**Published:** 2022-10-21

**Authors:** Ailing Liu, Jing Guo, Hua Liu, Yonghong Xu, Jun Wu, Tao Mao, Zibin Tian, Xueli Ding

**Affiliations:** a Department of Gastroenterology, the Affiliated Hospital of Qingdao University, Qingdao, China.

**Keywords:** benign intestinal stenosis, fibrosis, ulcerative colitis, ustekinumab

## Abstract

Intestinal stricture and obstruction are rare complications of ulcerative colitis (UC). Currently, there are only a few studies on the treatment of UC with intestinal stenosis, however there are no reports on the treatment of UC with benign intestinal stenosis with ustekinumab (UST).

**Patient concerns:**

A 22-year-old woman was admitted to our hospital due to a 3-year history of recurrent bloody mucous in stool with intermittent abdominal pain and distension developed in the past month. She was steroid-dependent and had developed a secondary loss of response to infliximab.

**Diagnoses:**

She was diagnosed with UC combined with incomplete intestinal obstruction due to stenosis. The stricture had a mixed pattern with both inflammatory and fibrotic components, with the former covering a larger section of the intestine.

**Interventions:**

The patient was given UST for 56 weeks.

**Outcomes:**

The patient’s symptoms subsided after treatment with UST. The ulcers healed, and the stenosis was reduced.

**Lessons:**

UST is effective against UC with benign intestinal stenosis. It is thought that UST inhibits the production of transforming growth factor-β and interleukin-17, leading to the suppression of myofibroblast proliferation, ultimately alleviating intestinal stenosis.

## 1. Introduction

Ulcerative colitis (UC) is a chronic, nonspecific inflammatory disease of the colon. The lesions usually start from the distal colon, develop reversibly, and are mainly limited to the mucosa and submucosa of the large intestine. Intestinal stricture and obstruction are rare complications of UC, and the underlying mechanism is still unclear. Ustekinumab (UST), which is an inhibitor of interleukin (IL)-12 and 23, was approved by the Food and Drug Administration in 2019 for the treatment of UC. UST reduces the number of active T cells, thereby preventing inflammation. Here, we report the first case where UC complicated with intestinal stricture was treated effectively by UST and discuss its mechanism of action.

## 2. Case report

**Figure 1.**
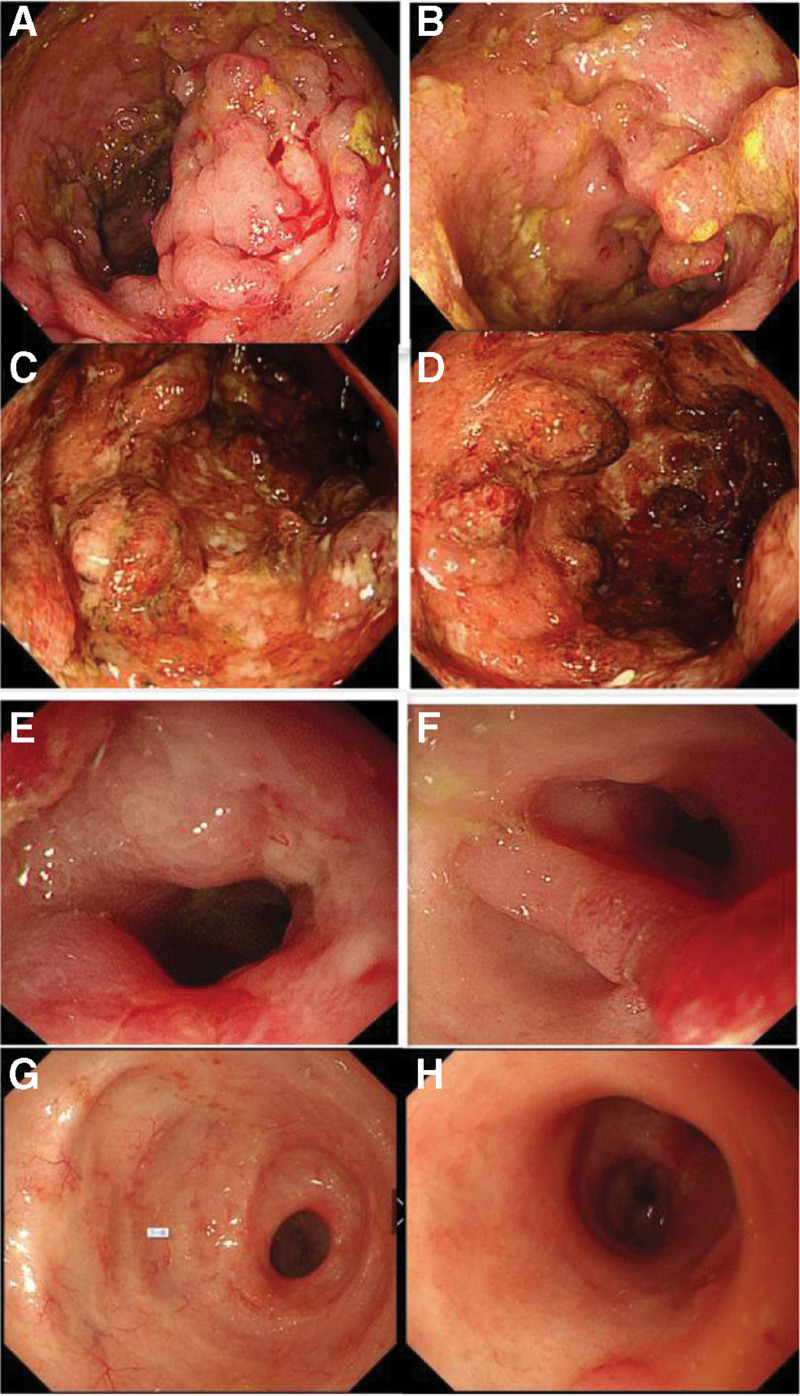
The entire colorectal mucosa was congested and swollen, with several inflammatory polyps. There were shallow ulcers in the ascending colon (A) and irregular large ulcers in the sigmoid colon (B). There was a stenose respectively in the transverse (C) and the descending colon (D). The mucosa were congested and swollen, with shallow ulcers and pseudopolyps. Cyclic ulcer with edema was observed in the sigmoid colon (E) and the endoscope was unable to pass due to the stenosis. The polypoid hyperplasia was seen at rectosigmoid junctions (F). The entire colorectal mucosa was scarred with inflammatory polyps in the descending (G) and sigmoid colon (H). The endoscope successfully passed through all the previously-stenotic sites.

A 22-year-old woman was admitted to our hospital in December 2020 due to a 3-year history of recurrent bloody mucous in her stool and a 1-month history of intermittent abdominal pain and distension. In August 2017, the patient started observing bloody mucous in her stool 10 to 15 times a day. The hemoglobin (HGB) was 56 g/L. Serum albumin (Alb) was 33.58 g/L. The C-reactive protein (CRP) level was 5.66g/L. Erythrocyte sedimentation rate (ESR) was 54 mm/h. Colonoscopy findings (Fig. 1A, B). The entire colorectal mucosa was congested and swollen with several inflammatory polyps. Shallow ulcers were observed in the ascending colon and irregular, large ulcers were found in the sigmoid colon. Endoscopic diagnosis was UC (E3, Mayo 3 points). The patient was treated with prednisone (30 mg qd po) and mesalamine (1 g qid po). When the stool returned to normal, prednisone dose was tapered and stopped after 3 months. Mesalazine (3–4 g/d) was used to maintain remission. In July 2018, the patient started observing blood and mucous in her stool again, this time 4 to 5 times per day, which was accompanied by fatigue and weight loss. HGB was 39 g/L, Alb was 30.68 g/L, ESR was 31 mm/h, and CRP was 5.36 g/L. Colonoscopy findings (Fig. 1C, D). Two stenoses were observed (one in the transverse and one in the descending colon). The stenoses allowed the passage of the endoscope. The mucosa was congested and swollen, with shallow ulcers and pseudopolyps. The patient was administered standard maintenance infliximab dosing(5 mg/kg) every 8 weeks after three induction doses at Weeks 0, 2, and 6. The consistency of the patient’s stool returned to normal, and its frequency decreased. However, after 54 weeks, the patient started observing mucous and blood in her stool again, this time 7 to 8 times a day. HGB was 62 g/L, and ESR was 51 mm/h. The infliximab level was low (1.2 µg/mL) and anti-infliximab antibody was negative. She was undertaken for treatment with 4-weekly infliximab from 62 weeks to 86 weeks. When her symptoms subsided, the patient was undertaken for treatment with 8-weekly infliximab from 94 weeks to 118 weeks. In December 2020, the patient developed diarrhea at a frequency of about five times a day with abdominal pain and distension. A physical examination found left lower abdominal tenderness without rebound pain or muscle tension. Bowel sounds were normal. The lower limbs were not swollen.

**Figure 2.**
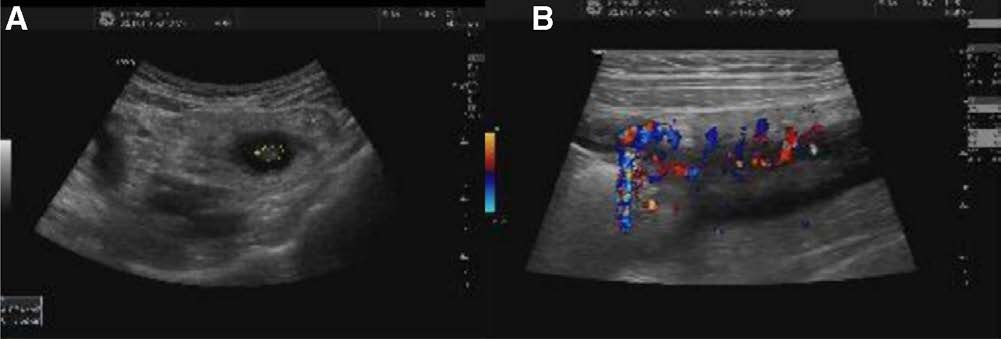
Intestinal wall thickening, echo reduction in the descending and the sigmoid colon was observed, the intestinal lumen was significantly narrowed (A), and the blood flow signal increased (B) as seen in the transabdominal ultrasound.

**Figure 3.**
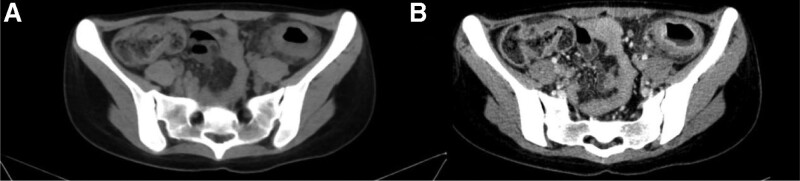
The descending and sigmoid colon narrowed (A) as seen in the abdominal CT. Colorectal wall thickening, mural enhancement and stratification is observed on the enhanced scan (B). CT = computed tomography.

After admission, the patient underwent thorough evaluations. HGB was 81 g/L, Alb was 28.25 g/L, ESR was 38 mm/h, and CRP was 9.17 g/L. Anti-neutrophil antibody was positive. CA-199 and CEA were negative. Transabdominal ultrasound (US) (Fig. 2) showed intestinal wall thickening, echo reduction and increased blood flow signal in the descending and sigmoid colon, and the intestinal lumen was significantly narrowed. Abdominal computer tomography (CT) (Fig. 3) showed colorectal wall thickening, mural enhancement and stratification on the enhanced scan. Narrowing of the descending and sigmoid colon was observed. Colonoscopy (Fig. 1E, F) showed cyclic ulcer with edema in sigmoid colon and the endoscope was unable to pass due to stenosis. Rectal mucosa was scarred, and polypoid hyperplasia was seen at rectosigmoid junctions.

The patient was diagnosed with UC with moderate activity, complicated by incomplete intestinal obstruction due to stenosis. The patient was switched to UST starting in January 2021. After 8 weeks of treatment with UST, her stool consistency had returned to normal, and the frequency was once a day. She had no abdominal pain or distension. From this point on, she was administered a subcutaneous injection of UST (90 mg) every 12 weeks.

During patient follow-up in March 2022 (56 weeks after starting UST treatment), the patient reported that she had no symptoms, and that her bowel movements had returned to normal, with one bowel movement a day. She gained 5 kg. HGB was 132 g/L, Alb was 44 g/L, ESR was 7 mm/h, and CRP was 0.37 g/L. There was no occult blood in her stool. Colonoscopy findings (Fig. 1G, H). The entire colorectal mucosa was scarred and there were inflammatory polyps. The endoscope passed through all of the previously stenotic sites.

## 3. Discussion

Here, we present the first effective treatment of UC with colon ulcers and stenosis using UST. The young patient with a 3-year history of recurrent UC presented with mucous and purulent stools, elevated inflammatory markers, anemia and hypoalbuminemia. She was steroid-dependent and had developed a secondary loss of response to infliximab. She developed intermittent abdominal pain and distension 1 month ago. Ultrasound, CT and colonoscopy showed sigmoid stricture. She was diagnosed with incomplete intestinal obstruction due to the stenosis.

The rate of stenosis varies from 1% to 11% in UC. UC patients with extensive colitis and long-standing disease can develop colorectal strictures. These are more often seen in patients with extensive colitis (17.1%) than those with left side colitis (7.5%).^[1]^ The mechanisms that induce inflammatory bowel disease-associated fibrosis may be related to excessive production of extracellular matrix by activated mesenchymal cells.^[2]^ Fibrostenosis in UC is much less common than it is in Crohn’s disease (CD). Whereas CD is a transmural disease, UC is limited to the mucosa. Therefore, stricture formation is much harder to understand and different mechanisms for stricture formation may exist in CD and UC.^[3]^ Yamagata et al^[4]^ have histopathologically examined and immunohistochemically stained the colon tissue samples of 9 stenotic and 17 non-stenotic UC patients. Their findings demonstrate a significant increase in basic-fibroblast growth factor-positive inflammatory cells and myofibroblasts in stenotic sections of the colon. This suggests that basic-fibroblast growth factor-positive neutrophils increase the proliferation of myofibroblasts, possibly resulting in fibrostenosis.

The clinical manifestations of UC complicated with intestinal stenosis were abdominal pain and distension, nausea, vomiting, abdominal bulge, loose stool, diarrhea, cessation of passage of stool or flatus. The US, CT, and magnetic resonance imaging are helpful for diagnosis.

Strictures are divided into two groups: inflammatory and fibrotic. In most instances inflammation and fibrosis coexist. The early stage of stenosis is inflammatory, which gradually progresses into fibrotic stricture. Inflammatory stenosis is due to intestinal congestion, edema and intestinal wall thickening, which can be alleviated by anti-inflammatory drugs. The intestinal US showed hypervascularization of the intestinal wall. CT showed intestinal wall thickening, mucosa stratification and mucosa enhancement. Prolonged exposure to injurious stimuli may lead to increased fibrotic stenosis. Currently, there are no widely approved anti-intestinal fibrosis drugs.^[4]^ Ultrasound elastography in patients with fibrous stenosis showed decreased tension in the intestinal wall tissue. Magnetic resonance imaging showed decreased T2 signal and reduced intestinal wall enhancement.^[5]^ According to the meta-analysis,^[6]^ the sensitivity and specificity for diagnosing small bowel inflammation in CD were 87% and 91% respectively with computed tomographic enterography, and 86% and 93% for magnetic resonance enterography. The US and CT of our patient showed intestinal wall thickening, increased blood flow signal, mucosal enhancement and mural stratification in descending and sigmoid colon. Therefore, the stricture had a mixed pattern with both inflammatory and fibrotic components, with the former accounting for a larger section of the colon.

Inflammatory bowel disease complicated with stricture is managed with medical, endoscopic, and surgical therapy. Corticosteroids and anti-tumor necrosis factor (TNF) agents can be used for inflammatory stenosis. If anti-inflammatory therapy is not successful, then endoscopic therapy or intestinal resection should be considered. Endoscopic approaches include endoscopic balloon dilatation, intra-lesion injection of corticosteroids or anti-TNF agents, and metallic biodegradable or removable stents.^[7]^ While there are a few studies on the treatment of UC with intestinal stenosis, there have been no attempts to treat UC with benign intestinal stenosis using UST. A thorough review by Lu et al^[8]^ indicates that anti-TNF agents are effective in the treatment of CD with intestinal stenosis by inhibiting inflammatory cell aggregation and reducing intestinal edema. However, data are lacking for UST and vedolizumab. Murate et al^[9]^ have reported that in two patients with CD, treatment with UST was effective for the management of small intestinal stenosis. UST suppresses the cytokine pathway of Th1 and Th17 cells by suppressing IL-23 and IL-12 production. Transforming growth factor (TGF-β) is the major player in intestinal fibrosis. Activated TGF-β promotes the differentiation from fibroblasts to myofibroblasts. Myofibroblasts produce collagen, fibronectin and α-smooth muscle actin.^[10]^ IL-17 up-regulates the production of collagen and tissue inhibitors of metalloproteinases.^[11]^ Both TGF-β and IL-17 can lead to extracellular matrix fibrosis and stenosis. UST could suppress myofibroblast proliferation and function, ultimately alleviating intestinal stenosis by decreasing TGF-β production in Th1 cells and reducing production of IL-22 and IL-17 by Th17 cells.^[9]^ Our patient, diagnosed with UC combined with intestinal stenosis, was symptom-free after 56 weeks of UST treatment. The ulcers had healed, accompanied by decreased stenosis.

## 4. Conclusions

Here we report the first case of effective treatment of UC complicated with intestinal obstruction due to benign intestinal stricture with UST. It is speculated that UST inhibits the production of TGF-β and IL-17. Our report provides promising information on the use of UST for UC with severe stenosis which could avoid early colectomy.

## Author contributions

**Investigation:** Yonghong Xu, Jun Wu.

**Writing – original draft:** Ailing Liu, Jing Guo, Hua Liu.

**Writing – review & editing:** Xueli Ding, Tao Mao, Zibin Tian.
